# Distribution of *Aedes* mosquito species along the rural–urban gradient in Lambaréné and its surroundings

**DOI:** 10.1186/s13071-023-05901-2

**Published:** 2023-10-12

**Authors:** Rodrigue Bikangui, Stravensky Terence Boussougou-Sambe, Mahmoudou Saidou, Barclaye Ngossanga, Ange Gatien Doumba Ndalembouly, Ynous Djida, Ayong More, Romuald Beh Mba, Haruka Abe, Yuri Ushijima, Steffen Borrmann, Bertrand Lell, Jiro Yasuda, Ayola Akim Adegnika

**Affiliations:** 1 Centre de Recherches Médicales de Lambaréné (CERMEL), BP 242 Lambaréné, Gabon; 2 École Doctorale Régionale d’Afrique Centrale de Franceville en Infectiologie Tropicale, Franceville, Gabon; 3 Institut Für Tropenmedizin, German Center for Infection Research (DZIF), Universität Tübingen, Tübingen, Germany; 4 Department of Emerging Infectious Diseases, Institute of Tropical Medicine (NEKKEN), Nagasaki University, Nagasaki, Japan; 5 Division of Infectious Diseases and Tropical Medicine, Department of Medicine 1, Medical University of Vienna, Vienna, Austria; 6 National Research Center for the Control and Prevention of Infectious Diseases (CCPID), Nagasaki University, Nagasaki, Japan; 7 Graduate School of Biomedical Sciences, Nagasaki University, Nagasaki, Japan; 8 Department of Parasitology, Leiden University Medical Center, 2333 ZA Leiden, The Netherlands; 9 Fondation Pour La Recherche Scientifique (FORS), BP 045 Cotonou, Benin

**Keywords:** *Aedes. albopictus*, *Aedes. aegypti*, *Hevea brasiliensis*, Rural area, Urban area, Lambaréné, Gabon

## Abstract

**Background:**

*Aedes albopictus* and *Aedes aegypti* are known for their potential as vectors of dengue (DENV) and chikungunya (CHIKV) viruses. However, entomological surveys are mostly carried out during epidemics. In Gabon where outbreaks of both viruses have occurred, there is no vector control program targeting these arboviruses. Therefore, we assessed the presence of *Aedes* species along a rural–urban gradient in Lambaréné (Gabon) and its surroundings and determined ecological factors associated to their presence.

**Methods:**

An entomological survey was conducted in Lambaréné and its surrounding rural areas. Mosquitoes were collected with aspirators around human dwellings, and ecological and environmental data were collected from each study area. Morphological identification keys were used to identify *Aedes* species. RNA was extracted from pools of female mosquitoes and amplified by RT-qPCR to detect the presence of DENV and CHIKV.

**Results:**

Overall, the most common vector collected was *Aedes albopictus (*97%, 4236/4367 specimens), followed by *Aedes aegypti* (3%, 131/4367)*. Albopictus* vectors was more abundant in the rural area (Wilcoxon signed-rank test*, Z* = *627, P* = 0.043) than in the urban area. In the urban area, a higher number of mosquitoes (45%) were recorded in the economic zone (zone 3) than in the historical and administrative zones (zone 1 and 2). In the rural area, the proportions of species numbers were significantly higher along the south rural transect (92%) compared to the north rural transect (Wilcoxon signed-rank test*, Z* = 43, *P *˂ 0.016). We also noted a high abundance of vectors in environments characterized by monocultures of *Hevea brasiliensis* (Hevea) and *Manihot esculenta *(cassava) (Kruskal–Wallis *H*-test*, H* = 25.7, *df* = 2, *P* < 0.001). Finally, no mosquito pools were positive for either DENV or CHIKV.

**Conclusion:**

*Aedes albopictus* was the dominant vector across the study sites due to its high invasiveness capacity. This presence re-affirms the potential for local transmission of both DENV and CHIKV, as indicated previously by serological surveys conducted in our study area, even though no transmission was detected during the current study. These findings underscore the need for regular arbovirus surveillance in the study region, with the aim of supporting vector control efforts in the event of outbreaks.

**Graphical Abstract:**

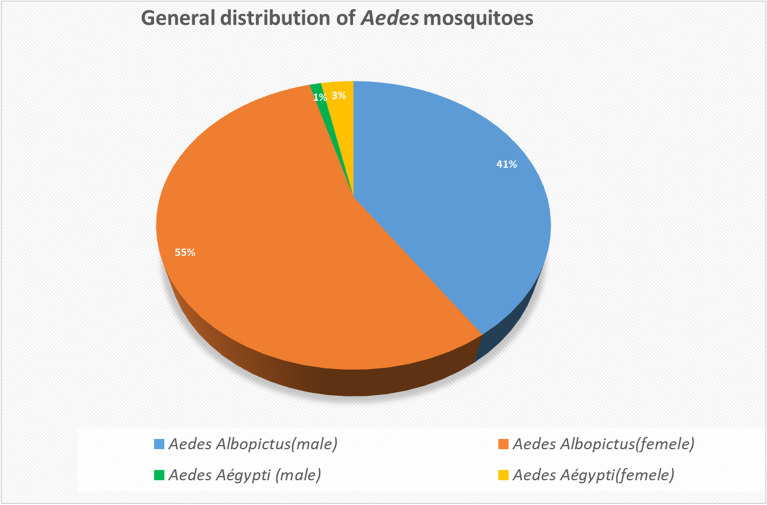

**Supplementary Information:**

The online version contains supplementary material available at 10.1186/s13071-023-05901-2.

## Background

Dengue and chikungunya are two widely distributed arboviral diseases that cause acute episodes characterized by symptoms such as fever and joint pain [1–3]. The major vectors of transmission are mosquitoes of the genus *Aedes*. Until recently, *Aedes aegypti* was considered to be the main epidemic vector for both dengue virus (DENV) and chikungunya virus (CHIKV) [4, 5], but in recent years the invasive species *Aedes albopictus* has been identified as the major vector during outbreaks in Asia and Africa [6]. *Aedes albopictus,* a species originating from Southeast Asia, was recently introduced in Africa, the Americas, Australia and Europe [7, 8] where in some cases it has gradually replaced the local species, *A*e*. aegypti* [9–11].

In Gabon, a country located in Central Africa, *Ae. albopictus* was first reported in 2006 in the city of Port-Gentil [12]. Its presence coincided with the occurrence of concomitant outbreaks of DENV serotype 2 (DENV2) and CHIKV. During these outbreaks, hundreds of patients were infected with both DENV2 and CHIKV in different towns of Gabon, with eight cases of co-infections reported [13–16]. *Aedes albopictus* was identified as the main vector species during this outbreak based on its higher prevalence compared to other vector species, such as *Ae. aegypti* and *Aedes simpsoni*, and the highest infection rates for both DENV2 and CHIKV, including one record of an *Ae. albopictus* positive for both CHIKV and DENV2 [17]. *Aedes albopictus* was also identified as the main vector species for CHIKV in a remote village of Gabon (Dangui). These studies demonstrated the spread of this mosquito species and its potential to drive arbovirus epidemics across the country [18].

Little data are available on these two arboviruses during inter-epidemic periods in Gabon, particularly in terms of the vectors involved in their transmission. Recent seroprevalence surveys in Moyen Ogooué province revealed a seroprevalence of 23% and 62% for DENV and CHIKV, respectively, among samples collected from 462 patients between 2014 and 2017 [19], while Lim et al. [20] reported a seroprevalence of 17.4% for DENV in Lambaréné for samples collected from 682 patients between 2015 and 2016. During this period, the presence of both DENV2 and DENV serotype 3 (DENV3) was confirmed by reverse transcription-quantitative PCR (RT-qPCR) in human samples [21].

These seroprevalences suggest the circulation of these arboviruses in the region and raise questions about the drivers of this transmission during non-epidemic periods. Assuming that DENV and CHIKV were preferentially transmitted by *Ae. albopictus* during past epidemics [22], we hypothesize that this vector is also responsible for the maintenance and circulation of viral strains in Moyen Ogooué province. Little information is currently available on the distribution of these vectors, or on the ecological factors favoring their expansion, thereby increasing the risk of future outbreaks, especially in the absence of vector control interventions against these vectors. This study therefore aims to assess the presence of *Aedes* mosquito species in Lambaréné and its surroundings to determine the ecological factors that influence their distribution range and the possible implication of these mosquito vectors in the spread of diseases.

## Methods

### Study areas

**Fig. 1:**
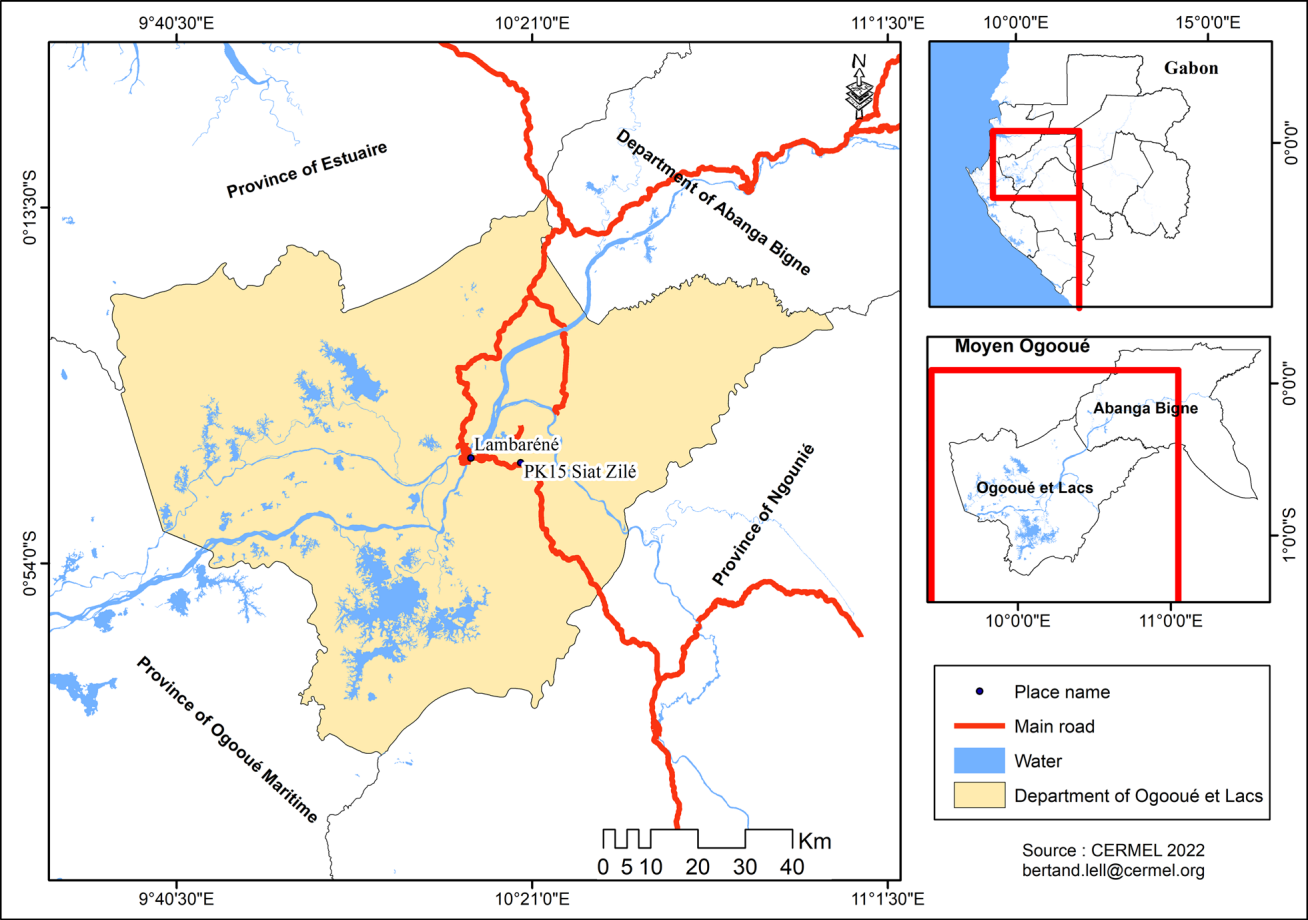
Map of Moyen Ogooué region showing the geographical location of Lambaréné

The study was carried out in different areas of Moyen Ogooué province which is located in the center of Gabon. The main city in the province is Lambaréné, located 250 km south of the capital Libreville, on the banks of the Ogooué river. Lambaréné has a population of 38,775 inhabitants [20] and is flanked by two major agricultural areas, with palm tree plantations 56 km north of the city and rubber tree plantations about 25–30 km south of the city (Fig. 1).

Gabon is subjected to four seasons annually, two dry and two rainy. Recently, however, changes in the climate have resulted in changes the rain patterns, with heavy rains recorded during months of dry seasons. Therefore, for this study we defined rainy seasons as those periods with monthly precipitation > 200 mm (October–December and March–June) and the dry seasons as periods with monthly rainfalls of < 200 mm (January–February and July–September). 

### Mosquito collection

The mosquitoes were collected in Lambaréné (urban) and its surroundings areas (rural) from November 2019 to March 2021 during repetitive cross-sectional surveys across multiple consecutive (wet and dry) seasons. Rural and urban areas were classified according to specific community conditions, such as the lack/absence (rural) or presence (urban) of public administrative structures and infrastructure. Lambaréné, the urban area, is the provincial capital with all administrative structures, including three referral hospitals, businesses, supermarkets, secondary-level schools, police stations, town hall, governor's office and modern water and electricity supply facilities. In contrast, the surrounding areas were defined as rural areas, characterized by the absence of almost all of the above mentioned structures and infrastructure.

In the rural area, collections were performed along two transects: Lambaréné-Makouké north towards Libreville (rural zone 1 [Rz1]) and Lambaréné—PK30 south towards Fougamou (rural zone 2 [Rz2]).

For the urban collections in Lambaréné, collection sites were divided into three zones separated by the natural physical barrier of the Ogooué river that divides the town into three distinct areas: zone 1 (historical zone, around the Albert Schweitzer museum); zone 2 (administrative zone, home to the main administration facilities); zone 3 (economic zone, characterized by trading and industrial activities). Each area in each zone was divided into neighborhoods. Capture sessions were performed in each village and neighborhood in areas covering a radius of 50 to 100 m and identified as conducive to the presence of *Aedes* spp. according to information reported in the literature (presence of gutters, trees, shaded areas in the vicinity of human dwellings).

Mosquitoes were collected using electrical aspirators (Rule 4″ In-Line Blower, 240 V, 7.0 amperes). The collections were carried out in open air in the morning from 6:00 a.m. to 10:00 a.m. in a discontinuous manner around human dwellings by two collectors who generally were separated from each other by 10 to 20 m. The capture points were not fixed in the area, and the collectors captured mosquitoes within the pre-defined radius. Mosquitoes were caught either sitting on the foliage of flowers and grasses or flying around the collector. Microhabitats with shade and dead leaves on the ground were favored for the capture of mosquitoes. All mosquitoes collected within one collection session by each collector were grouped in the same cage with access to a 10% sugar solution and then transferred to the Medical Entomology Laboratory of the Centre de Recherches Médicales de Lambaréné (CERMEL). Mosquito collections were carried out at least twice per week at each study site. Geographic information system (GIS) data were recorded for each collection site using the Global Positioning System (GPS) Essentials GPS tool (version 2.0). The geographical coordinates (latitudes and longitudes) of the recorded areas were exported in GPX 1.1 format and saved in QGIS software for mapping.

Ecological and environmental data were also collected, including information on habitat, presence of water access points, vegetation type, type of crops, breeding site, presence of fruit trees (such as wild apples and mangoes on which mosquitoes feed on sugar when the fruit is ripe), human activity and climatic conditions during the collection.

### Identification of *Aedes* vectors

The mosquitoes were identified morphologically using a Zeiss Stemi 508 stereo microscope equipped with a binocular lens (Carl Zeiss AG,Oberkochen, Germany)) and the identification keys from Leopoldo [23]. All collected mosquitoes identified as *Aedes* spp. were grouped into separate pools of 15–20 male and female *Ae. aegypti* or *Ae. albopictus* mosquitoes. These mosquito pools were transferred into 2-ml Eppendorf tubes (Eppendorf AG, Hamburg, Germany) containing RNAlater solution (Thermo Fisher Scientific, Waltham, MA, USA) and stored at − 80 °C until further processing 

### Viral RNA extraction and detection by RT-qPCR

Female mosquitoes of both *Aedes* spp. were homogenized using the protocol from Frentiu et al. [24] with a slight modification. The pools of *Aedes* spp. were transferred into tubes containing 2-mm beads (Lysing Matrix Z; MP Biomedicals, Santa Ana, CA, USA) and ground in phosphate-buffered saline (PBS) a FastPrep-24™ 5G homogenizer (MP Biomedical). RNA was then extracted using the Qiagen RNA Mini Kit following the manufacturer’s instructions (Qiagen, Hilden, Germany).

We used previously described primer and probe sequences from Santiago et al. [25]. For the detection of DENV, we performed a multiplex RT-qPCR that detects the four DENV serotypes (DENV1, DENV2, DENV3, DENV4). RT-qPCR was also used for the detection of CHIKV. The RT-qPCR reactions were performed in a 20-µl reaction mixture using a 2× One-Step PrimeScript RT-PCR kit (Takara Bio., Kusatsu, Japan). Each reaction mix contained 10 µl of 2× One-Step PrimeScript RT-qPCR Mix, 10 µM of Primer Probe mix (primer plus probe), 0.2 µl of a passive Rox fluorochrome, pure water without RNase and 2 µl of RNA template. The RT-qPCR was performed using a LightCycler 480 Instrument II PCR platform (Roche Applied Science, Penzberg, Germany) at cycling conditions of 5 min at 52 °C; 10 s at 95 °C and 45 cycles of 5 s at 95 °C and 35 s at 60 °C. Samples with cycle threshold (Ct) values ≤ 40 were considered to be positive.

### Statistical analysis

The overall distribution of vectors was determined by the frequencies of the different vectors in the study area using the formula % = (*A*/ N) × 100, where *A* is the number of *Aedes* mosquitoes per site collected and *N* is the total population of vectors collected.

The Capture Effort Index (CEI), defined as the number of mosquitoes caught per collector per hour (m/c/h), was used to determine vector abundance. This index was calculated over four parameters, including *A* (number of *Aedes* mosquitoes per site), *F* (frequency of site visits), *C* (number of collectors) and *D* (duration of each collection session according to the following formula: CEI = *A*/(*F* × *C* × *D*). The difference in vector abundance was assessed by comparing the median CEI in each zone or environment using the Wilcoxon signed-rank test and Krustal-Wallis test. Interquartile range (IQR) was taken into consideration to account for the asymmetric distribution of our data in the study. As the data collected were count data, we performed a multiple linear regression to determine the association between various ecological factors (access to water, vegetation type, type of crops, breeding site and fruit tree) on the outcome (abundance of vectors) in each study area. All analyses were performed using R software, and the significance level was set at *α* ˂ 0.05.

## Results

A total of 4367 *Aedes* mosquitoes were collected in the study areas. The number of mosquitoes collected ranged from two to 1042 by site. The median number of mosquitoes collected was 50. *Aedes albopictus* represented approximately 97% (4236/4367) of vectors collected, with the remaining 3% (131/4367) comprising *Ae. aegypti*.

### Vector distribution by CEI in the study areas

*Aedes albopictus* was present in roughly equal proportions in the rural and urban areas, with CEI of 110.5 m/c/h in the rural area and CEI of 122 m/c/h in the urban area (Table 1). The median CEI of *Ae. albopictus* by collection site in the rural area (3.6 m/c/h, IQR 0.2–9.9 m/c/h) differed significantly from that in the urban area (0.5 m/c/h, IQR 0–3.5 m/c/h) (Wilcoxon signed-rank test, *Z* = 627, *P* = 0.043); in contrast, *Ae. aegypti* was only found in urban areas.

**Table 1:** Distribution of *Aedes* species across the study areas

Study areas/zones	*Aedes* spp.		*Aedes albopictus*			*Aedes aegypti*		
	Total *N* (%)	Total CEI	CEI	Median (IQR)	Statistic test	CEI	Median (IQR)	Statistic test
*Study areas*								
Rural	2028 (47)	110.5	110.5	3.6 (0.2–9.9)	Z = 627, *P* = 0.043	0	0 (0–0)	*Z* = 406, *P* = 0.15
Urban	2323 (53)	124.9	122	0.5 (0–3.5)		3	0 (0–0)	
*Urban zones*								
LBN-zone 1 (historical zone)	493 (21)	13.6	10.8	0.33 (0–0.8)	*H* = 1.65, *df* = 2, *P* = 0.44	3	0 (0 -0.2)	*H* = 324, *df* = 2, *P* ˂ 0.001
LBN-zone 2 (administrative zone)	796 (34)	46.7	46.6	0.9 (0–4.4)		0.1	0 (0–0)	
LBN-zone 3 (economic zone)	1034 (45)	64.6	64.6	1.5 (0–4.8)		0	0 (0–0)	
*Rural zones*								
PK-LBN-route south FGM (Rz2)	1871 (92)	100.7	100.7	10 (8–17)	*Z* = 43, *P* ˂ 0.016	0	0 (0–0)	-
PK-LBN-route north LBV (Rz1)	157 (7.7)	9.8	9.8	0 (0–2)		0	0 (0–0)	

*CEI* Capture Effort Index, *FGM* Fougamou,* IQR* inter-quartile range, * LBN* Lambaréné,* LBV* Libreville,* PK* route PK30,* Rz1*,* Rz2* rural zones 1, 2

In the rural area, the collection of *Aedes* mosquitoes along the transects (zones) where the collections were carried out revealed that there was a higher abundance of *Ae. albopictus* (CEI 100.7 m/c/h) in villages located in Rz2 compared to Rz1 (CEI 9.8). *Aedes albopictus* vectors were significantly more abundant (Wilcoxon signed-rank test, *Z* = 43, *P *˂ 0.016) in Rz2 (median 10 m/c/h, IQR 8–17 m/c/h) than in Rz1 (median 0 m/c/h, IQR 0–2 m/c/h). The distribution of *Ae. albopictus* along these two transects was heterogeneous, with this vector more prevalent in villages such as Zilé for the Lambaréné-PK30-Fougamou transect (Rz2) and Bindo for the Lambaréné-Makouké transect (Rz1).

In the three distinct zones of the urban area, the distribution of *Aedes* species showed that urban zone 3 (economic) had the highest CEI of *Ae. albopictus* (CEI 64.6 m/c/h) followed by urban zone 2 (CEI 46.6 m/c/h) and then by urban zone 1 (CEI 10.8 m/c/h) (Table 1). Despite this difference, the abundance of *Ae. albopictus* in these three zones was essentially the same, with no statistically significant difference (Kruskal–Wallis *H*-test, *H* = 1.65, *df* = 2, *P* = 0.44) between urban zone 3 (median 1.5 m/c/h, IQR 0.0–4.8 m/c/h) compared to urban zone 2 (median 0.9 m/c/h, IQR 0.0–4.4 m/c/h) and urban zone 1 (median 0.3 m/c/h, IQR 0.0–0.8 m/c/h).

**Fig. 2:**
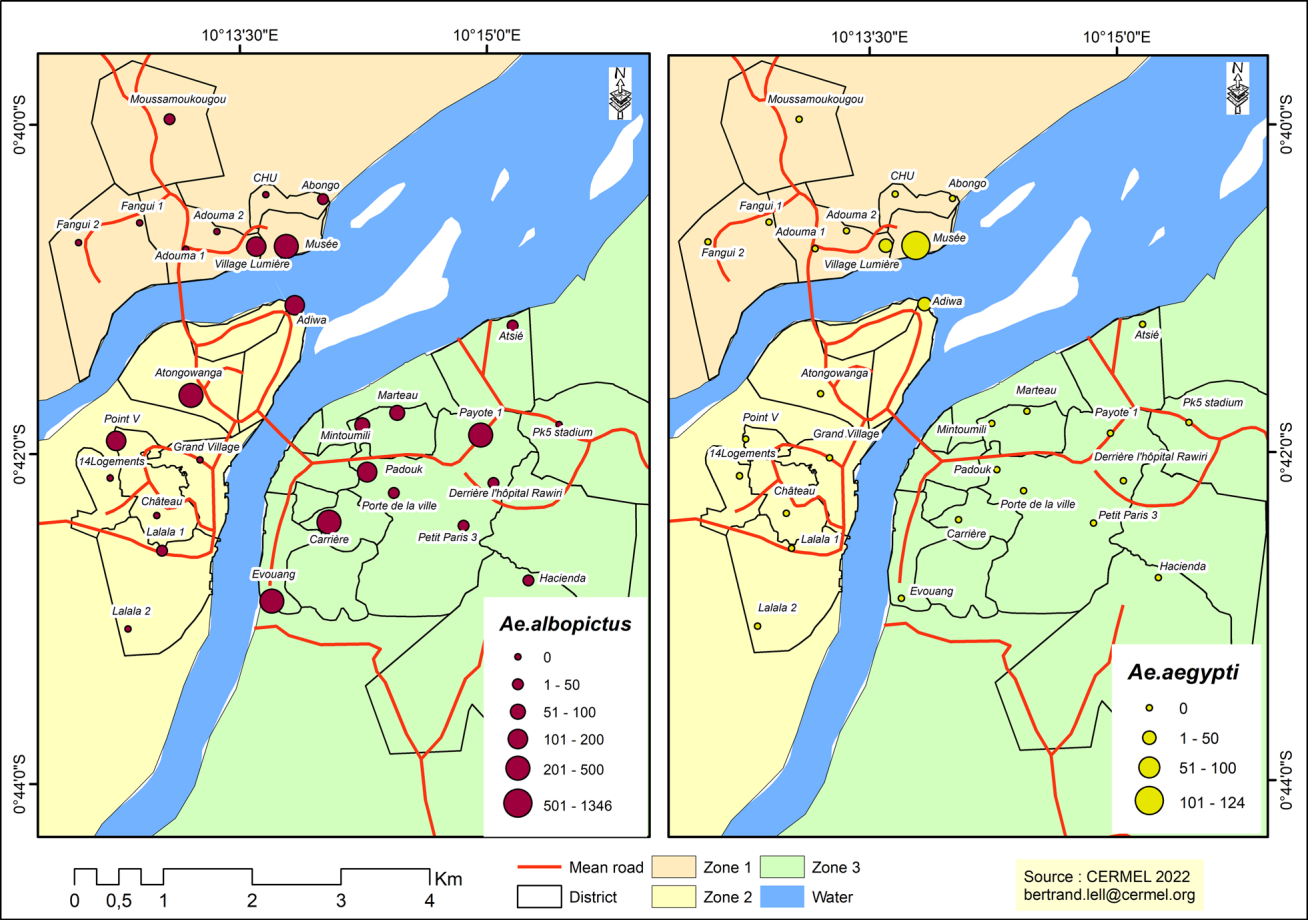
Distribution of *Aedes albopictus* (left) and *Aedes aegypti* (right) caught from sampling sites in urban areas

Figure 2 shows the spatial distribution of vectors in the three different urban zones. *Aedes albopictus* vectors were clearly more abundant in urban zone 3 with at least four neighborhoods (Carriere, Evouang, Paillote, Padoouk) in which a minimum of 201 mosquitoes were collected by site. However, two other neighborhoods, in urban zone 2 and 1, respectively, had comparable mosquito numbers. Although poorly represented, *Ae. aegypti* mosquitoes were mainly found in the Museum (*n* = 124) district in urban zone 1 and sporadically in the surrounding districts (Lumière) with fewer individuals (*n* = 6).

**Fig. 3:**
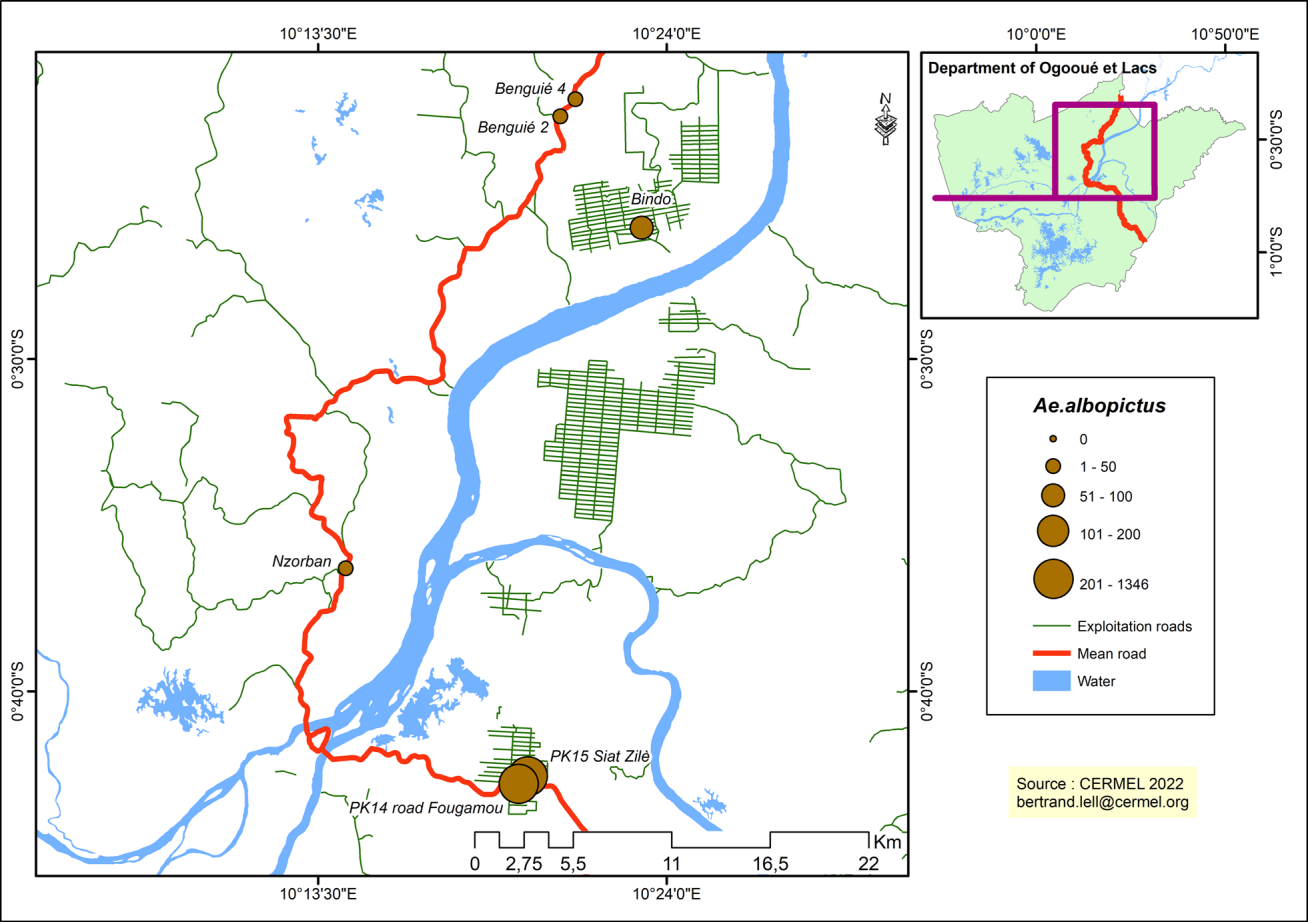
Distribution of *Aedes albopictus* in rural zones

The data reported in Fig. 2 also show that the spread of *Aedes* mosquitoes in the city remains heterogeneous although, importantly, sites with high recorded numbers of mosquitoes were located in areas near the Ogooué river and its tributaries. By contrast, in rural areas (Fig. 3), the spread of *Ae. albopictus* vector populations was localized in specific agricultural sites, such as rubber plantations along the Lambaréné-PK30-Fougamou transect (Rz2), with a minimum of 501 mosquitoes by collection site.

### Influence of ecological factors on the abundance of vectors

The distribution of vectors was also evaluated according to a set of defined ecological and environmental factors, such as season, access to various types of water sources, type of vegetation, crops grown, presence of fruit trees and presence and quality of breeding sites.

*Aedes* mosquitoes were collected in both the dry (CEI 87.9 m/c/h) and rainy seasons (CEI 182.4 m/c/h) (Table 2). Despite the high CEI in the rainy season, there was no difference in the abundance of *Ae. albopictus* between seasons (Wilcoxon signed-rank test*, Z* = 837.5, *P* = 0.47), with a comparable median number of *Ae. albopictus* captured during the dry season (CEI: median 1.0 m/c/h, IQR 0.0–5.6 m/c/h) and the rainy season (CEI: median 0.5 m/c/h, IQR 0.0–3.5 m/c/h). Similarly, for *Aedes aegypti*, no difference between seasons, (Wilcoxon signed-rank test*, Z* = 706, *P* = 0.3) was observed.

**Table 2:** Assessment of vectors distribution and abundance according to ecological factors

Ecological factors	*Aedes* spp.		*Ae. albopictus*			*Ae. aegypti*		
	Total* N* (%)	Total CEI	CEI	Median (IQR)	Statistic test	CEI	Median (IQR)	Statistic test
*Seasons*								
Dry	1432 (33)	87.9	87.8	1.0 (0–5.6)	*Z* = 837.5, *P* = 0.47	0.1	0 (0–0)	*Z* = 706, *P* = 0.3
Rain	2919 (67)	182.4	179.2	0.5 (0–3.5)		3.1	0 (0–0)	
*Access to water*								
Rain	2358 (33)	131.2	131.2	0.6 (0–6.8)	*H* = 2.36, *df* = 3, *P* = 0.5	0	0 (0–0)	*H* = 455, *df* = 3, *P* < 0.01
Tap	2105 (29)	111.3	108.4	2.1 (0.4–4.7)		3	0 (0–0)	
River	2551 (35)	143.2	143.2	0.4 (0–5.9)		0	0 (0–0)	
Wells	215 (3)	13.4	13.4	0.0 (0–3.7)		0	0 (0–0)	
*Vegetation type*								
Herbaceous	2003 (41)	104.9	102	0.8 (0–4.4)	*H* = 21, *df* = 3, *P* < 0.001	3	0 (0–0)	*H* = 580, *df* = 3, *P* < 0.04
Plantation	1871 (38)	100.7	100.7	9.8 (8–17)		0	0 (0–0)	
Tropical forest	715 (15)	28.5	28.5	0.1 (0–3.3)		0	0 (0–0)	
Urban forest	281 (5.8)	17.6	17.6	0.0 (0–0)		0	0 (0–0)	
*Type of crops*								
* Hevea brasiliensis*	1761 (37)	97.3	97.3	14.5 (9.1- 31)	*H* = 25.7, *df* = 2, *P* < 0.001	0	0 (0–0)	*H* = 464, *df* = 3, *P* = 0.2
* Manihot esculenta*	1778 (37)	93.9	93.8	5.0 (3.1–7.5)		0.1	0 (0–0)	
No cultivated crops	1210 (25)	56.4	53.5	0.3 (0 -2.0)		2.9	0 (0–0)	
*Presence of fruit trees*								
No	1926 (44)	115.8	114.4	0.0 (0–2.1)	*Z* = 559, *P* = 0.05	1.4	0 (0–0)	*Z* = 792, *P* = 0.5
Yes	2425 (56)	119.6	118.1	1.2 (0–5)		1.6	0 (0–0)	
*Presence of breeding site*								
No	837 (19)	51.3	51.3	0.1 (0 -3.1)	*Z* = 620.5, *P* = 0.10	0	0 (0–0)	*Z* = 644, *P* < 0.05
Yes	3514 (81)	184.2	181.2	0.9 (0–5.3)		3	0 (0–0)	
*Status of breeding site*^*a*^								
Negative	686 (20)	29.8	26.9	0.2 (0–0.8)	*Z* = 107.5, *P* < 0.001	2.9	0 (0–0.6)	*Z* = 349, *P* < 0.06
Positive	2828 (80)	154.4	154.3	5.0 (1.1–8.4)		0.1	0 (0–0)	

*CEI* Capture Effort Index,* IQR* inter-quartile range

^a^Negative or positive status means absence or presence, respectively, of larvae in the breeding site

Table 2 show a significant difference in the abundance of *Ae. albopictus* collected across areas with different vegetation types, with the median CEI of *Ae. albopictus* collected in areas with rubber fields being higher than the median CEIs of *Ae. albopictus* from areas with wild forest, urban forest and herbs or shrubs (Kruskal–Wallis *H*-test, *H* = 21, *df* = 3, *P* < 0.001). The median CEI of *Ae. albopictus* collected was statistically different according to the types of crops grown in the areas where the mosquitoes were collected (Kruskal–Wallis *H*-test, *H* = 25.7, *df* = 2, *P* < 0.001). Specifically, the median CEI of mosquitoes was higher in areas where the Brazilian rubber tree *Hevea brasiliensis* (14.5 m/c/h, IQR 9.1–31 m/c/h) was the main crop grown compared to areas where the dominant crop was cassava *Manihot esculenta* (5 m/c/h, IQR 3.1–7.5 m/c/h) or where there was no agriculture (0.3 m/c/h, IQR 0.0–2 m/c/h).

Although the number of *Ae. albopictus* collected tended to be higher in areas where the main source of water supply was rainwater (2374/4367; 33%) than in areas where the main water access was either tap water, river or wells, the difference was not statistically significant overall (Kruskal–Wallis *H*-test, *H* = 2.36, *df* = 3, *P* = 0.5). Likewise, the median CEI of *Ae. albopictus* was significantly higher in areas with fruit trees compared to an environment without fruit trees (Wilcoxon signed-rank test*, Z* = 559, *P* = 0.05). Moreover, no difference in abundance of *Ae. albopictus* mosquitoes was observed in sites with or without the presence of breeding sites (Wilcoxon signed-rank test*, Z* = 620.5, *P* = 0.10). However, the presence of positive Culicidae breeding sites significant influenced the median CEI of *Aedes* mosquitoes collected (2.5 m/c/h, IQR 0.9–6.7 m/c/h) in the study area (Wilcoxon signed-rank test*, Z* = 107.5*, P* < 0.001).

Regarding the association of *Ae. aegypti* and the ecological and environmental factors, no conclusion on the results can be provided. The low values of the CEI and the calculated median CEI linked to an extremely low number and a localized distribution only in zone 1 of the urban area of the vector did not allow us to draw a conclusion, despite the* P*-values reported in Table 2.

### Associations between ecological factors and vector abundance

The impact of ecological factors on the abundance of *Ae. albopictus* mosquitoes in the study areas was assessed. Table 3 reports the results of the linear regression obtained from the analysis of six out the seven pre-determined ecological factors. For most of these factors, with the exception of those related to the presence of fruit trees (regression coefficient [*β*] = − 1.69) and well water (*β* = − 0.29), *β* was positive, although not statistically significant. Access to water showed no association with vector abundance.

**Table 3:** Factors associated with the abundance of *Aedes albopictus *mosquitoes

Ecological predictors	Number of observations	Estimate of regression coefficient *β*	95% Confidence interval	*P*-value
*Access to water*				
Tap	44	1	–	–
Rain	26	2.51	[− 2.0, 7.0]	0.274
River	33	1.81	[− 2.4, 6.0]	0.397
Wells	6	− 0.29	[− 8.3, 7.7]	0.943
*Vegetation typ*e				
Urban forest	20	1	–	–
Herbaceous	49	1.26	[-2.0, 4.6]	0.448
Rubber field	6	15.9	[10, 22	< 0.001
Tropical forest	20	0.55	[− 3.4, 4.5]	0.783
*Type of crops*				
No cultivated crops	50	1	–	–
* Hevea brasiliensis*	4	23.19	[17, 30]	< 0.001
* Manihot esculenta*	21	3.34	[0.18, 6.5]	0.038
*Presence of fruit trees*				
No	29	1	–	–
Yes	52	− 1.69	[− 5.3, 1.9]	0.346
*Presence of breeding sites*				
No	33	1	–	–
Yes	48	2.28	[− 1.2, 5.7]	0.192
*Status of breeding site*^*a*^				
Negative	25	1	–	–
Positive	23	5.52	[0.02, 11]	0.049

^a^Negative or positive status means absence or presence, respectively, of larvae in the breeding site

In contrast, regarding factors related to vegetation type, only rubber tree forests were associated with vector abundance (linear regression, *β* = 15.9, *P* ˂ 0.001). Similarly, in the crop-related factor group, *Manihot esculenta* (linear regression*, β* = 3.34, *P* = 0.038) and *Hevea brasiliensis* (linear regression*, β* = 23.19, *P* ˂0.001) were strongly associated with *Ae. albopictus *abundance in the study area.

Finally, although there was no association between the abundance of adult mosquitoes and the presence of breeding sites, we found an association between abundance of mosquitoes and status of breeding sites in the study area (linear regression, *β* = 5.52, *P* = 0.049).

### Characterization of DENV and CHIKV in *Aedes* species collected

A total of 122 pools of adult female *Ae. albopictus* and *Ae. aegypti* mosquitoes, totaling approximately 2240 mosquitoes, were extracted and screened by RT qPCR. The presence of neither DENV nor CHIKV was detected in any of the mosquito pools.

## Discussion

Regular surveillance of potential arbovirus vectors and assessment of ecological factors that play a role in their distribution and spread are important for designing control strategies that are tailored to local settings.

The entomological surveys based on the collection of adult mosquitoes carried out in this study allowed us to identify *Ae. albopictus* as the most aggressive species and potentially the most abundant vector in rural areas, with *Ae. aegypti* found in low numbers and only in urban areas of Lambaréné. This predominance of *Ae. albopictus* is in line with collections carried out in Libreville and Lastrouville during the DENV and CHIKV outbreaks in 2007 and 2010, respectively, during which this species identified as the main vector of the infections [14, 18, 26]. These results indicate the expansion and establishment of *Ae. albopictus* in many countries of the Central African region, to the detriment of *Ae. aegypti*, believed to have originated in Africa, as the main vector of various arboviruses [6, 18, 27–30]. Satyrization, which is a form of reproductive competition in which males of one species mate with female of another species, resulting in the production of less-fit hybrid offspring, could explain the progressive replacement of *Ae. aegypti* by the recently introduced *Ae. albopictus* [31]*.* In addition, interspecific competition for resources during larval stages can sometimes contribute to the displacement of some species to the detriment of another. Previous studies showed that sympatry between these two main vectors in the urban environment contributes to the displacement of *Ae. aegypti* [32, 33], as previously reported in Cameroon and the Central African Republic [34]. In the present study,* Ae. albopictus* was not evenly distributed across all zones, with larger numbers collected in urban zone 3, where commercial and industrial activities such as markets are concentrated. The proximity of this area to Rz2 as well as the availability of suitable breeding sites could further explain the abundance of *Ae. albopictus* in such environments [35].

Although *Ae. albopictus* was present in all environments, the survey also showed that these mosquitoes were more abundant along the Lambaréné-PK30-Fougamou transect (Rz2). This transect is home to a vast scheme of rubber tree plantations that may provide suitable conditions for the development of *Aedes* larvae. In particular, harvesting pots used for the collection of the rubber sap are potential breeding sites for *Aedes* species and contribute to the maintenance of *Aedes* spp. populations in this environment, which further points to an anthropological role in setting up favorable conditions for vector development [1]. On the other hand, despite the presence of a palm-oil plantation scheme on the Lambaréné–Bindo–Libreville transect, fewer *Aedes* were collected in this area. Therefore, the presence of rubber crops appears to be a major driver for the development of *Aedes* mosquitoes in the rural area studied. Similar results were reported in an entomological survey assessing the effect of changes in land-usage on the distribution and abundance of vectors in environments dominated by oil palm (*Elaesis guineensis*), cocoa and cassava plantations in the southern part of the Ivory Coast [36].

Several studies have demonstrated the role of environmental conditions in the development and invasion of vectors [37]. The presence of larval breeding sites, the influence of seasons, humidity, type of vegetation, presence of fruit trees and the influence of crops are some of the factors investigated in our survey, with the aim to determine their association with vector abundance. We found no difference in the distribution of vectors between the dry season and the rainy season. The rainfall recorded (Additional file 1: Fig. S1) during these dry periods, although lower compared to the rainy season, provides the necessary conditions for vector development.

The survey data (Additional file 2: Dataset) also provided insight into the influence of breeding sites and their status on the presence and abundance of *Aedes* mosquitoes. Our results show that the presence of these breeding sites and their positivity is weakly associated with the abundance of vectors in the area, as often described in studies where distribution and abundance were assessed on the basis of mosquito egg collection [35, 38]. In contrast to these earlier studies, our collections consisted of adult mosquitoes, which could explain the weak association we observed, in addition to the fact that culicine larvae were not reared to the adult stage and therefore not identified specifically as *Aedes*.

It also appears from this survey that the vectors have a specific tropism for a type of vegetation and for a certain type of crop, as already reported by Zahouli [36] in the Ivory Coast, indicating that *Aedes* spp. mosquitoes were widely abundant in polycultures in which the Euphorbiaceae family was highly represented. In our survey, several collections were made in areas with *Manihot esculenta* crops, including urban zone 3 and part of urban zone 2 where a relatively higher number of *Aedes* mosquitoes were collected.

Finally, no viral carriage was found from the pools of mosquitoes analyzed. This is surprising if we consider the high seroprevalences of 20.4% found by Yuri and collaborators in the Lambaréné population [19] where we found a high abundance of *Ae. albopictus*. This result could be explained by the fact that the collections in our study were performed during the non-epidemic period when viral carriage and the circulation of strains may be low; higher numbers of virus-carrying vectors would be expected when there is an active and intense circulation of the viruses in the human population [14, 18]. The viral carriage reported in the *Ae. albopictus* mosquitoes during the epidemic period of 2007 to 2010 in Gabon was obtained by collecting mosquitoes in and around the houses of people found to be infected with arboviruses [13]. A similar approach could be used in surveys during inter-epidemic period by performing collections in the surroundings of people with a non-malaria fever or those who are seropositive for arboviruses.

One limitation in this study was the use of mosquito vacuums, such as those used in our collections. The power of the hoover may result in the disappearance of some of the distinctive features of the vector of interest, as the suction airflow may be intense during collection. This could be a confounding factor when discriminating between two vectors that are morphologically close and thus create an under- or over-estimation of one or the other vector.

## Conclusion

Integrated vector control strategies against the transmission of arboviruses and other vector-borne diseases are an important public health priority for Gabon. In the present study, we have provided evidence identifying *Ae. albopictus* as the most widespread potential arbovirus vector in Moyen Ogooué province. Some of the ecological factors listed in this survey to be associated with the vector population demonstrate the important role of humans in the proliferation of this species in rural and urban areas. Activities associated with industrial rubber plantations to the south of Lambaréné, as well as those situated in two other areas of Gabon (Kango and Mitzic), are potential hotspots for the invasion of these vectors into other areas, as highlighted in this study.

### Supplementary Information

**Additional file 1: Fig S1.** Rainfall data on two years, November 2019 to April2021 to Lambaréné.

**Additional file 2. Dataset.** Entomological survey in Lambaréné and its surroundings.

## Data Availability

All data supporting the conclusions of the study are included in the manuscript and its supplementary information.

## Abbreviations

CEI: Capture Effort Index
CHIKV: Chikungunya virus
DENV: Dengue virus
RT-qPCR: Reverse transcription-quantitative PCR
