# Supplementary figures and images for: Distribution of Aedes mosquito species along the rural–urban gradient in Lambaréné and its surroundings
Source: Parasit Vectors. 2023 Oct 12;16:360. doi: 10.1186/s13071-023-05901-2 (PMC10571480; doi:10.1186/s13071-023-05901-2)

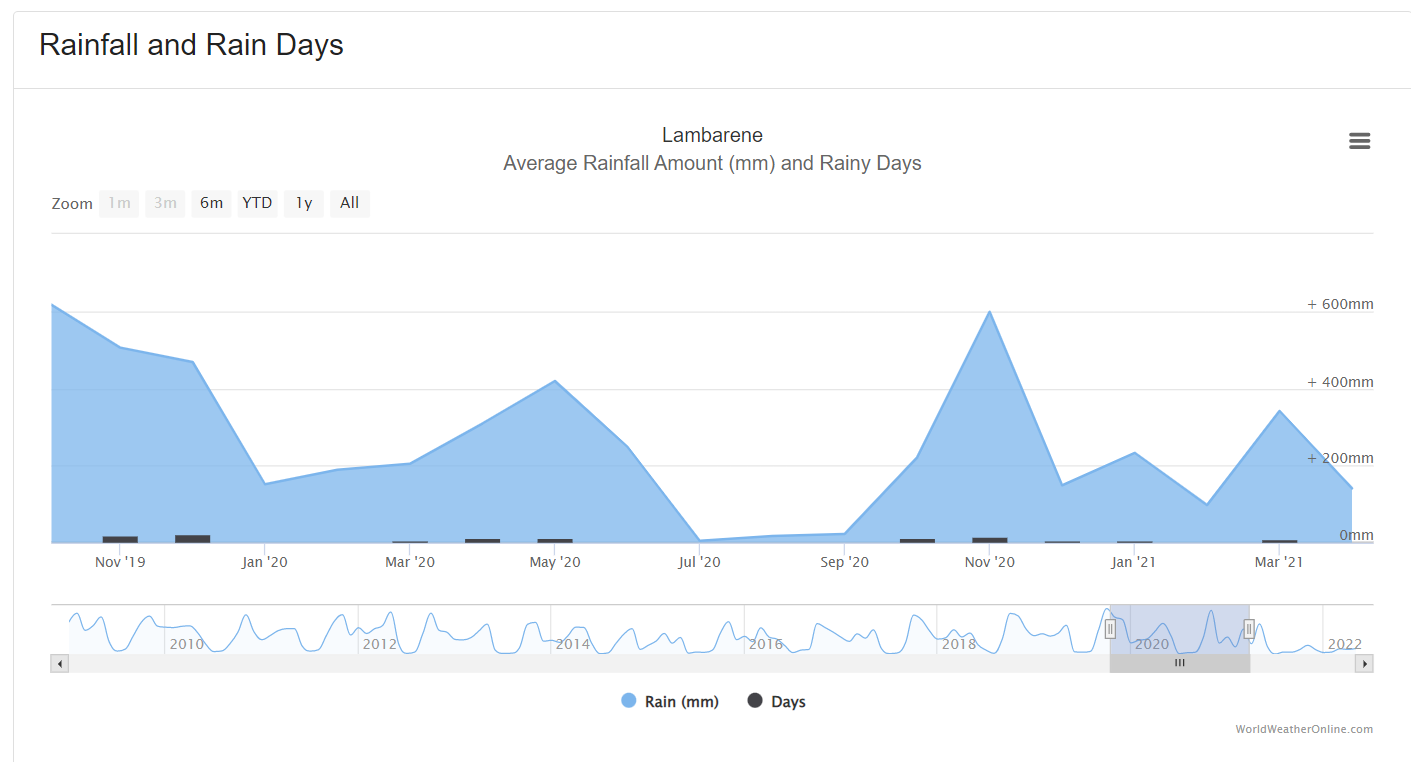

Supplement: Supplementary file 1 — Additional file 1: Fig S1. Rainfall data on two years, November 2019 to April2021 to Lambaréné. [file 13071_2023_5901_MOESM1_ESM.tif]
